# DeepRespNet: a hybrid attention-recurrent framework for non-contact respiratory rate estimation

**DOI:** 10.3389/fphys.2026.1792689

**Published:** 2026-05-20

**Authors:** Sreya Deb Srestha, Uday Debnath, Sungho Kim

**Affiliations:** Department of Electronic Engineering, Yeungnam University, Gyeongsan-si, Republic of Korea

**Keywords:** BiLSTM, CNN, noncontact, remote photoplethysmography, respiration rate, rPPG

## Abstract

**Introduction:**

The noncontact measurement of respiratory rate (RR) has gained considerable attention recently due to its relevance to remote healthcare and continuous physiological monitoring. However, existing camera-based approaches often exhibit reduced accuracy in subjects having darker skin tones, primarily owing to melanin absorption and variations in illumination that degrade remote photoplethysmographic (rPPG) signal quality. This study aims to develop a robust deep learning framework that ensures reliable RR estimation across diverse skin tones.

**Methods:**

A hybrid deep learning framework, referred to as DeepRespNet, is proposed that jointly analyzes rPPG and motion signals extracted from facial and thoracic regions in RGB video sequences. The core feature encoder, termed RespFormer, integrates spatiotemporal convolution with multi-head self-attention to capture both local and long-range respiratory patterns. The learned representations are further processed by a separate bidirectional long short-term memory (BiLSTM) network to model temporal coherence and generate physiologically stable respiratory waveforms. Optical-flow-based motion features and rPPG color variations are combined to form a multi-channel respiratory representation.

**Results:**

The proposed framework was evaluated on a multi-subject dataset with synchronized reference respiration signals. Experimental results achieve mean absolute errors of 0.45 breaths per minute (BPM) for light-skinned subjects and 0.80 BPM for dark-skinned subjects. Bland–Altman and cross dataset analyses further confirm strong agreement and consistent performance across skin tone groups.

**Conclusion:**

The proposed framework enables reliable and skin-tone-aware noncontact respiratory rate estimation. Initial findings indicate its potential suitability for camera-based respiratory monitoring in remote healthcare and telemedicine applications, though further validation on larger populations is required.

## 1 Introduction

Respiratory rate (RR) is a vital physiological parameter and an important early indicator of respiratory distress, sleep apnea, and other cardiorespiratory conditions (Maharaj et al., 2015; Rambaud-Althaus et al., 2015). Traditional methods of RR monitoring, such as chest belts, spirometry, or capnography, provide reliable measurements but typically require contact-based sensors. These can be uncomfortable, may restrict movement, and are unsuitable for long-term or remote monitoring applications, especially in telemedicine settings (Gwak et al., 2023). Over the past decade, non-contact approaches to respiratory monitoring have emerged as promising alternatives. These methods can be classified into one of two categories. The first category is remote photoplethysmography (rPPG), or camera-based PPG, which derives physiological signals from color variations in the skin captured via RGB camera (Bauer et al., 2008). The other category comprises motion-based approaches, which extract respiratory signals by tracking subtle chest, torso, or facial motion using optical flow, motion magnification, or feature tracking.

The rPPG-based estimation of RR and heart rate (HR) has been widely studied, and accurate cardiorespiratory monitoring can be achieved by using standard cameras under controlled conditions (Mateu-Mateus et al., 2021; Kashevnik et al., 2021). These methods leverage variations in blood volume pulse linked to physiological rhythms, thereby enabling noncontact measurement at a distance. Chrominance-based approaches, such as the CHROM method, have shown enhanced robustness to motion and illumination compared with blind source separation (BSS) techniques (de Haan and Jeanne, 2013). Similarly, a new method named GRGB rPPG employs linear RGB transformations to improve performance under indoor lighting and motion conditions (Haugg et al., 2023). Moreover, BSS methods like ICA and PCA, along with envelope-based fitting, have improved pulse morphology reconstruction in noisy environments, emphasizing the importance of advanced signal processing for robust rPPG estimation (Sun et al., 2023).

However, despite these strengths, rPPG has certain limitations. One of the drawback is its sensitivity to skin pigmentation, as melanin concentration tends to attenuate optical signals and results in reduced signal-to-noise ratios (SNRs) for darker-skinned individuals. Several studies have reported a measurable difference in performance across Fitzpatrick skin types, raising concerns about demographic fairness (Addison et al., 2018; Dasari et al., 2021). Meta-analyses further confirm that imaging based PPG techniques consistently underperform for the darker skin tones (Nowara et al., 2020). More recent investigations on publicly available rPPG datasets have identified persistent higher error rates in darker-skinned subjects, highlighting structural bias in existing benchmarks (Bondarenko et al., 2025). Additionally, Diverse R-PPG (Chari et al., 2020) have demonstrated that while HR estimation can be evaluated across a wide range of skin tones, performance disparities remain significant. To overcome these drawbacks, approaches such as PhysFlow (Comas et al., 2024) have introduced skin-tone-aware augmentation strategies to improve HR estimation robustness. However, these studies primarily focus on HR estimation, and the influence of skin tone variation on camera-based RR estimation has not been significantly investigated, highlighting a critical research gap addressed in this work.

There are widely used public benchmark dataset for physiological signal estimation, including PURE (Stricker et al., 2014), UBFC-rPPG (Bobbia et al., 2019), COHFACE (Heusch et al., 2017), VIPL-HR (Niu et al., 2018), and MAHNOB-HCI (Soleymani et al., 2011), which primarily focus on blood volume pulse (BVP) and HR estimation, with limited emphasis on RR estimation as mentioned in **Table 1**. Therefore, this study introduces a custom dataset aimed at addressing these gaps by focusing on RR estimation across diverse skin tones.

**Table 1:** Available public datasets.

Dataset	No. of subjects	Physiological signal	Ground truth sensor
PURE [Stricker et al., 2014]	10	BVP, HR, SpO_2_	CMS50E pulse oximeter
UBFC-rPPG [Bobbia et al., 2019]	42	BVP, HR, SpO_2_	CMS50E Pulse oximeter
VIPL-HR [Niu et al., 2018]	107	BVP, HR, SpO_2_	CONTEC CMS60C BVP sensor
MANHOB-HCI [Soleymani et al., 2011]	27	ECG, RR	CONTEC CMS60C BVP sensor
COHFACE [Heusch et al., 2017]	40	BVP, RR	Respiration belt
TokyoTech rPPG [Maki et al., 2019]	9	BVP, HR	ProComp Infiniti T7500M
MMSE-HR [Rapczynski et al., 2019]	40	HR, RR, Blood pressure	Biopac Mp150 BVP Sensor
MMPD [Tang et al., 2023]	33	BVP, HR	Finger PPG (HKG-07 C+) at 30 H

In motion-based respiratory estimation, optical flow and motion magnification techniques are used to capture subtle thoracic displacements to monitor breathing patterns. These methods perform effectively under low illumination or partial occlusion, as they rely on geometric motion cues rather than variations in pixel intensity (Pediaditis et al., 2022; Ahani et al., 2024). Recent studies have integrated optical flow with deep spatiotemporal networks, enabling stable respiration tracking across different poses and lighting conditions (Manne et al., 2023). Such approaches are particularly beneficial when rPPG quality is poor, such as for subjects with darker skin tones, as they provide a reliable, motion-driven alternative to noncontact respiratory monitoring.

Despite these strengths, motion-based approaches also face notable limitations. Basic optical flow is highly sensitive to non-respiratory body movements, camera jitter, or background interference, which often introduces noise into the breathing signal (Gwak et al., 2022). Motion magnification techniques, although effective at amplifying subtle respiratory movement, can also unintentionally enhance irrelevant motion or noise, particularly in recordings by a handheld or unstable camera, unless additional stabilization techniques are applied (Alam et al., 2017).

Recently, deep learning-based approaches have gained attention for RR estimation by integrating data-driven feature extraction with the modeling of temporal and spectral dynamics. Convolutional neural networks (CNNs) effectively learn the spatial representations of skin pixels, improving robustness against illumination changes and noise compared to traditional signal processing techniques (Shihab, 2025). Recurrent models such as long short-term memory (LSTM) networks capture long-term temporal dependencies, enhancing the accuracy of cycle-level predictions in noisy sequences (Lin et al., 2019). Attention mechanisms further refine these models by adaptively emphasizing informative temporal and spatial features, thereby improving generalization to real-world conditions (Chen et al., 2024). Moreover, generative architectures, including adversarial and CycleGAN-based frameworks, have been explored to reconstruct respiratory components from weak or corrupted signals, demonstrating strong adaptability under low signal-to-noise conditions (Aqajari et al., 2021).

While deep learning has shown strong potential, most existing approaches face limitations that affect reliability and generalization. Many rely on a single region of interest (ROI), typically the face or chest, making them susceptible to signal degradation caused by posture changes, occlusions, or localized motion artifacts (Romano et al., 2021; Yu et al., 2019a). Systems that use facial regions alone often fail under masks, hair occlusion, or non-frontal poses (Speth et al., 2022). Additionally, skin tone variability remains underexplored, even though higher melanin concentration absorbs a larger portion of the incident visible light. This reduces the amount of reflected light available for rPPG extraction, weakening the optical signal and potentially affecting performance across demographic groups (Dasari et al., 2021; Bondarenko et al., 2025). Finally, while deep learning has shown strong potential in contactless HR estimation (Chen and McDuff, 2018), its integration into RR estimation remains limited and has prevented current models from fully leveraging both physiological priors and data-driven learning (Park and Hong, 2023).

To address these limitations, this study develops a unified learning framework that jointly analyzes rPPG and motion-derived cues from facial and thoracic regions. The framework first employs a lightweight RespFormer module, which integrates spatio-temporal convolution with multi-head self-attention to capture both local and long-range respiratory patterns and enhance respiration-related feature representations. These features are then processed by a bidirectional long short-term memory (BiLSTM) network, which models temporal dependencies in the frame sequence and enables accurate reconstruction of the respiratory waveform. The key contributions of this study are as follows:

- A novel RespFormer feature encoder that integrates convolutional and multi-head attention mechanisms to effectively capture spatiotemporal respiratory cues from RGB-derived signals.
- A bidirectional temporal modeling module using BiLSTM to improve cycle-level coherence and enhance the stability of estimated respiratory waveforms.
- A dual-ROI strategy incorporating both facial rPPG and thoracic motion signals to improve robustness under challenging illumination and subject variability.
- A skin-tone-aware evaluation protocol that analyzes performance across light and dark skin groups, providing insights into the fairness and cross-demographic consistency of the proposed method.

This manuscript is organized into three main sections. The Materials and Methods section describes the proposed framework, including signal extraction, preprocessing, the hybrid RespFormer-BiLSTM architecture, and experimental setup. The Results section presents performance metrics, comparative analysis, and validation across skin tone groups. The Discussion section interprets findings, addresses limitations, and outlines future research directions.

## 2 Material and methods

**Figure 1:**
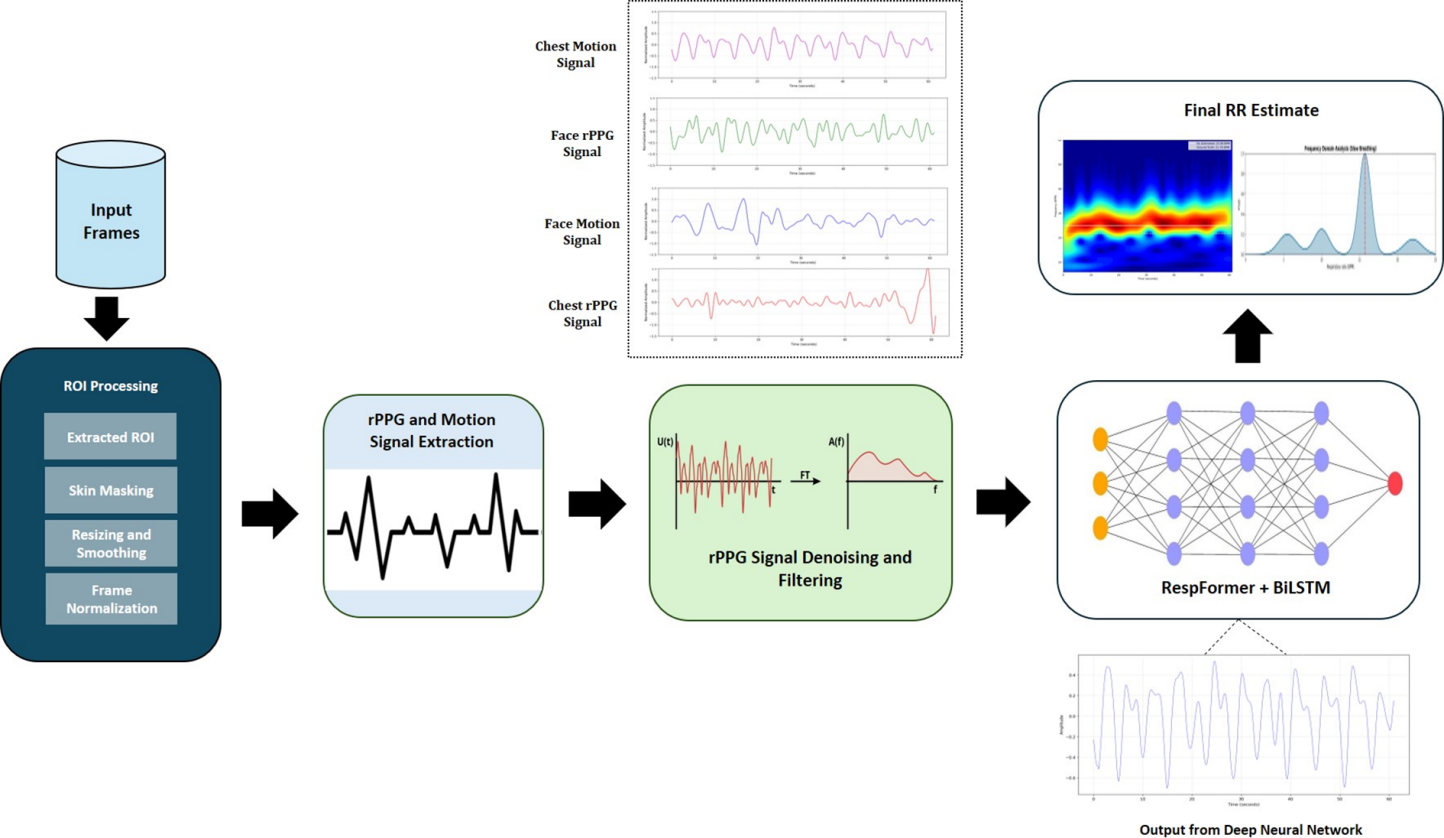
Overall workflow of the DeepRespNet framework.

This work proposes a noncontact RR estimation framework using RGB video as the primary input. Each frame provides high-resolution spatial and color information across the red, green, and blue channels, thereby enabling reliable ROI detection and tracking. The system integrates facial and chest ROIs as complementary sources, with facial regions offering rPPG signals and chest regions encoding respiratory motion. The overall processing pipeline is illustrated in **Figure 1**.

### 2.1 Experimental setup and data collection

**Figure 2:**
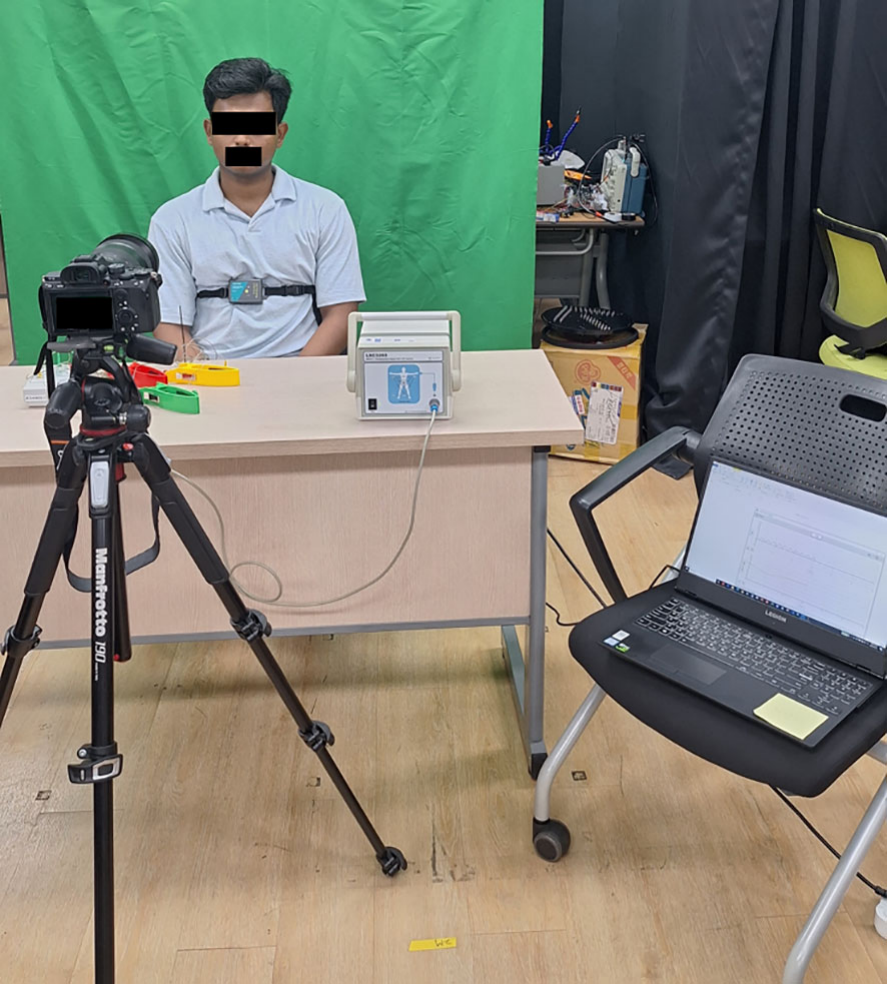
Overview of the data acquisition setup showing camera position, subject posture, and the placement of the reference respiration belt.

The experimental data were collected using a Sony Alpha 7 III camera recording at a resolution of 6000 × 4000 pixels and 30 fps. The camera was positioned 1 m from each subject (**Figure 2**) to maintain consistency in framing and image quality. Each recording captured both the facial and upper chest regions to represent respiration-induced motion under ambient lighting conditions of approximately 500 lux, measured using a digital lux meter. A uniform green background was used to reduce visual noise, whereas other environmental conditions remained natural. Participants were seated comfortably and instructed to breathe normally without motion restrictions, ensuring realistic acquisition conditions.

The dataset consisted of 28 participants (14 male, 14 female) from diverse ethnic groups, including South Asian, Middle Eastern, and East Asian populations, all aged between 25 and 62 years (mean ± SD: 28.71 ± 8.37 years). The recorded respiratory rates ranged from 6 to 30 BPM, covering slow to normal resting breathing patterns. Participants were categorized into five skin tone groups (types I–V) based on the Fitzpatrick skin type scale. Classification was performed through visual inspection of facial and forearm skin under controlled indoor illumination conditions. This classification represents a subjective visual grouping for analytical purposes rather than a clinical dermatological diagnosis. Consistent with prior work in camera-based physiological monitoring (Talukdar et al., 2023), participants were subsequently divided into lighter (types I–II, n= 17) and darker (types III–V, n= 11) skin tone groups for comparative analysis. This study was conducted in accordance with the Institutional Review Board (IRB) regulations as per approval, and also informed consent forms were collected prior to the experiment. This diversity enabled an evaluation of model consistency across varying skin tones and facial characteristics, though the sample size limits broader generalization claims. Each recording session lasted for 60 s.

Ground-truth respiratory signals were simultaneously recorded using a Go Direct Respiration Belt (Code: GDX-RB), worn around the thorax (**Figure 2**). The sensor output was recorded in CSV format for synchronization and comparison with the non-contact respiratory signals extracted from the proposed framework.

Since the camera and respiration belt operate as independent acquisition systems, temporal synchronization was performed in the post-processing step by matching their dominant breathing cycles using cross-correlation analysis. The time delay corresponding to the maximum cross-correlation between the two signals was used to determine the temporal offset, after which a constant temporal shift was applied to the camera-derived signal to align it with the belt reference. Prior to alignment, both signals were resampled to the same temporal resolution based on the camera frame rate.

All preprocessing, signal processing, and model implementation were developed in Python 3.12. Video processing and region extraction were implemented using OpenCV and NumPy, signal processing operations including filtering and cross-correlation were performed using SciPy, and the deep learning components were implemented in PyTorch (version 2.9.1).

### 2.2 ROI extraction

The initial step involves acquiring RGB video sequences of the subject, followed by the detection of facial and chest regions—two areas exhibiting respiration-induced and blood volume pulse motion. Facial detection was performed using the Haar cascade classifier (Viola and Jones, 2001), selected for its computational efficiency and real-time capability on resource-constrained devices. The classifier computes Haar-like features as intensity differences between adjacent rectangular regions, as defined in Equation 1:

(1) $$f(x)=\sum_{i\in R_{\text{white}}}I(i)-\sum_{j\in R_{\text{black}}}I(j)$$

where *I*(*i*) denotes the pixel intensity at location *i*, *R*_white_ and *R*_black_ represent the white and black rectangular regions in the Haar kernel, respectively. After the facial bounding box (*x,y,w,h*) was detected, the location of the chest ROI was geometrically inferred by projecting a second region directly below the face. The vertical and horizontal boundaries of the chest ROI were defined in Equations 2 and 3, respectively.

(2) $$y_{\text{chest},\text{ start}}=y+h,\;y_{\text{chest},\text{ end}}=y+\alpha h$$

(3) $$x_{\text{chest},\text{ start}}=x-\beta w,\;x_{\text{chest},\text{ end}}=x+(1+\beta )w$$

where *α* and *β* are scaling factors that determine the vertical and horizontal extent of the chest region relative to the detected face. The values were set to *α* = 2.5 and *β* = 0.2, determined empirically across all subjects in the dataset to identify the configuration that most consistently captured the upper thoracic region across varying body sizes and camera distances. The resulting ROI extends approximately 2.5*h* below the top of the face bounding box and is expanded horizontally by 20% of the face width on both sides. These proportional factors were selected to reliably includes the region where respiratory motion is most prominent, while maintaining spatial consistency across subjects.

Skin segmentation was then performed using dual color-space thresholds in the YCrCb and HSV domains, ensuring illumination invariance. In the YCrCb space, skin pixels were identified based on chrominance ranges of the *Cr* and *Cb* channels, while in the HSV space, thresholds were applied to the hue and saturation components. The corresponding binary masks were defined in Equations 4 and 5, respectively.

(4) $$M_{\text{ycrcb}}=\{\begin{array}{ll} 1, & \text{if }Cr_{\text{min }}\leq Cr\leq Cr_{\text{max }}\;\text{and}\;Cb_{\text{min }}\leq Cb\leq Cb_{\text{max }}\\ 0, & \text{otherwise} \end{array}$$

(5) $$M_{\text{hsv}}=\{\begin{array}{ll} 1, & \text{if }H_{\text{min }}\leq H\leq H_{\text{max }}\;\text{and}\;S_{\text{min }}\leq S\leq S_{\text{max }}\\ 0, & \text{otherwise} \end{array}$$

where *Cr*, *Cb*, *H*, and *S* denote the chrominance and hue-saturation components of each pixel, and the thresholds (*Cr*_min_*,Cr*_max_*,Cb*_min_*,Cb*_max_) and (*H*_min_*,H*_max_*,S*_min_*,S*_max_) define the skin-color ranges in the respective color spaces. The threshold ranges were adopted from previously reported skin-detection models in the YCrCb and HSV color spaces (Chai and Ngan, 1999), which characterize typical skin chrominance and hue-saturation distributions across varying illumination conditions.

Pixels satisfying either condition formed the final skin mask, as expressed in Equation 6:

(6) $$M_{\text{skin}}=M_{\text{ycrcb}}\vee M_{\text{hsv}}$$

**Figure 3:**
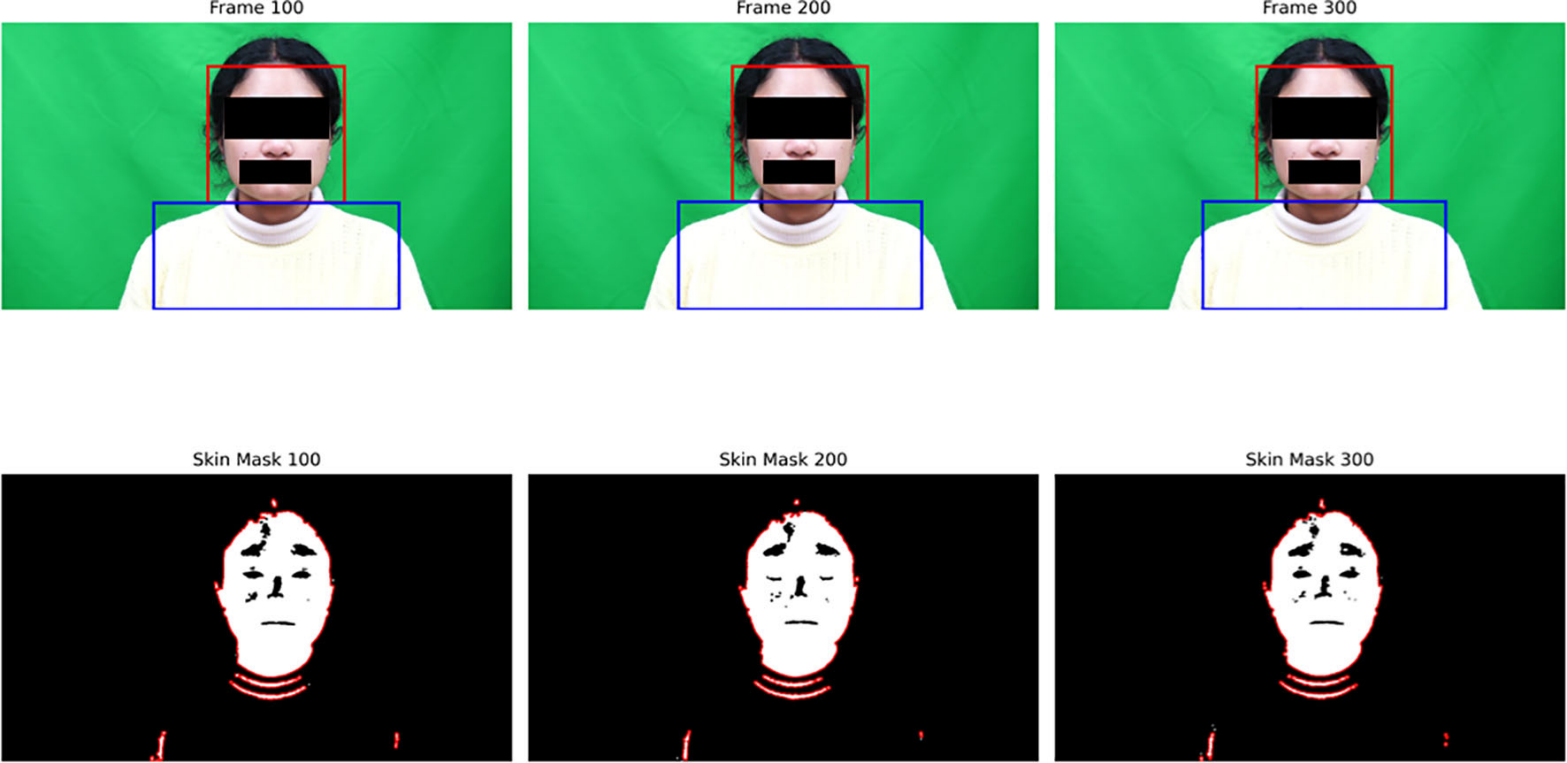
Illustration of ROI extraction and skin masking across frames. The upper row shows detected face (red) and chest (blue) ROIs, and the lower row shows corresponding binary skin masks highlighting skin-reflective regions.

The resulting mask was further refined using Gaussian smoothing and morphological filtering to suppress noise and small isolated regions (Saryanto and Andriyani, 2024). The final face and chest ROIs were extracted using Equations 7.

(7) $$ROI_{\text{face}}=I\cdot M_{\text{skin},\text{ face}},\;ROI_{\text{chest}}=I\cdot M_{\text{skin},\text{ chest}}$$

where *I* denotes the original RGB frame. This ensures that only skin-reflective regions are preserved, minimizing interference from background or clothing. **Figure 3** illustrates this process, showing the detected ROIs and corresponding binary skin masks across consecutive frames. This dual-ROI configuration effectively captures plethysmographic variations from the face (Verkruysse et al., 2008) and respiration-induced motion from the chest, thereby providing a reliable mechanical coupling of breathing dynamics (Cheng et al., 2023).

### 2.3 ROI processing

After the face and chest ROIs were extracted, a preprocessing stage was applied to enhance signal reliability for subsequent analysis. Non-skin pixels identified during segmentation were masked to zero intensity, thus ensuring that only skin-reflective regions contributed to feature computation. To suppress high-frequency noise from illumination flicker, sensor variability, or minor pixel motion, temporal smoothing was applied using a Gaussian filter. For each pixel intensity sequence *x*(*t*), a Gaussian kernel *G_σ_*(*t*) was convolved as defined in Equation 8.

(8) $$x_{\text{smooth}}(t)=(x*G_{\sigma })(t)=\sum_{\tau =-k}^{k}x(t-\tau )\cdot \text{exp }(-\frac{\tau^{2}}{2\sigma^{2}})$$

where *σ* controls the smoothing bandwidth and *k* denotes the kernel half-width. This step effectively removed rapid fluctuations while preserving low-frequency components corresponding to respiratory modulations of 0.1–0.6 Hz.

In addition, frame normalization was applied to minimize inter-subject variability in skin tone and illumination. For each frame *I_f_*, pixel intensities were rescaled is defined in Equation 9.

(9) $$I_{f,\text{norm}}=\frac{I_{f}-\mu_{f}}{\sigma_{f}}$$

where $\mu_{f}$ and $\sigma_{f}$ denote the per-frame mean and standard deviation of pixel values within the skin mask, respectively. These steps standardized and denoised the ROIs, thereby ensuring uniform feature quality across varying skin tones (Talukdar et al., 2023).

### 2.4 Signal processing

From the pre-processed ROIs, two complementary physiological signals were derived to capture respiration-related variations, an rPPG-based signal and a motion-based respiratory component. The rPPG signal was obtained by averaging green-channel intensities across skin pixels, as the green band provides the highest signal-to-noise ratio (SNR) for capturing subtle blood volume variations (Verkruysse et al., 2008). Simultaneously, respiration-induced motion was estimated using the Lucas–Kanade optical flow algorithm (Queiroz et al., 2020), with vertical displacements weighted toward thoracic regions to emphasize breathing dynamics. These two signal modalities capture distinct and complementary physiological relevence.

Both signals were first detrended to remove slow baseline variations caused by illumination changes and sensor drift. The detrended signals were then bandpass-filtered (0.1–0.6 Hz) using a fourth-order Butterworth filter to suppress residual low-frequency drift and high-frequency noise while preserving respiratory oscillations. To avoid phase distortion, the filter was implemented using a zero-phase forward–backward filtering procedure. The Butterworth filter response is defined in Equation 10:

(10) $$H(s)=\frac{1}{\sqrt{1+{\left(\frac{s}{\omega_{c}}\right)}^{2n}}},\;n=4$$

where $\omega_{c}$ represents the cutoff frequency and *n* is the filter order.

Following the filtering, signals were normalized using a three-sigma rule to ensure comparability across subjects having different skin tones. The normalization is expressed in Equation 11.

(11) $$x_{\text{norm}}(t)=\frac{x(t)-\mu_{x}}{3\sigma_{x}}$$

where x(t) is the input signal, $\mu_{x}$ is the temporal mean, and $\sigma_{x}$ is the temporal standard deviation of the signal.

To facilitate temporal analysis, overlapping windows of 200–300 frames were extracted with 80%–90% overlap, balancing temporal resolution and data continuity. To further investigate the relative contribution of different ROIs, a weighted fusion of face and chest signals was analyzed using Equation 12:

(12) $$S(t)=0.8\cdot S_{\text{chest}}(t)+0.2\cdot S_{\text{face}}(t)$$

where $S_{\text{chest}}(t)$ and $S_{\text{face}}(t)$ are the chest and face signals, respectively. A higher weight was assigned to the chest component due to its stronger physiological coupling and superior SNR than the face signal (Schrumpf et al., 2019). A grid search over different weight combinations was performed to evaluate how these regions contribute to respiratory representation (**Table 2**).

**Table 2:** Ablation study on signal fusion weights.

Chest weight	Face weight	MAE (Light)	MAE (Dark)
0.5	0.5	0.72	1.15
0.6	0.4	0.61	1.02
0.7	0.3	0.53	0.91
0.8	0.2	0.45	0.80
0.9	0.1	0.52	0.88
1.0 (chest only)	0.0	0.58	0.95

MAE values are reported in BPM. Bold values indicate the selected configuration. The 0.8/0.2 weighting achieves optimal performance by leveraging the high-SNR chest signal while retaining complementary facial information.

**Figure 4:**
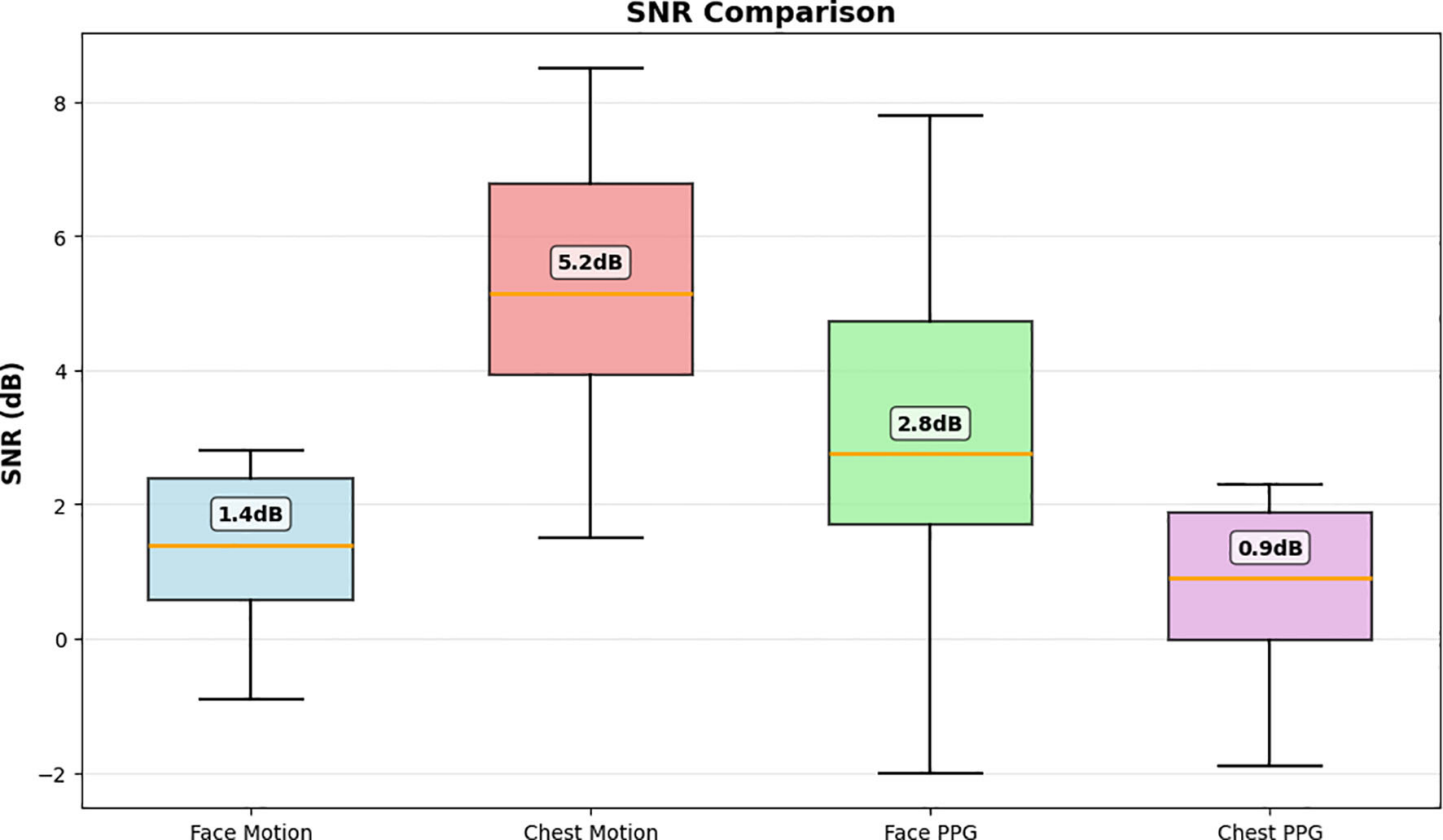
SNR comparison of respiration signals extracted from face and chest ROIs. Chest motion exhibits the highest reliability, which justifies the weighted fusion scheme.

This analysis (**Figure 4**) shows that chest motion signals exhibit higher SNR value, indicating stronger and more stable respiratory patterns. In contrast, facial rPPG signal exhibits lower SNR value, however, it captures complementary physiological variations influenced by blood perfusion. These findings suggest that while chest motion provides a dominant respiratory signal, rPPG signals from facial regions provide additional physiological information that enhances robustness under varying skin tones and environmental conditions. Overall, this signal processing strategy highlights the physiological relevance of combining multiple ROIs, demonstrating that both regions provide complementary insights of the respiratory activity.

### 2.5 DeepRespNet architecture

The proposed DeepRespNet framework is designed to fuse motion- and color-based physiological cues from two key ROIs: the face and the chest. From each region, an rPPG signal and a motion signal are extracted, and these produce a four-channel temporal input. These channels are first synchronized, normalized, and then transformed into a unified tensor of shape (Batch, Time, 128), where the final dimension corresponds to the projected feature embedding. The model processes this tensor using a hybrid architecture composed of a RespFormer encoder, which integrates convolutional feature extraction with multi-head self-attention, and a BiLSTM module to capture both local and long-range respiratory dynamics. The following subsections describe each component of this architecture. .

#### 2.5.1 RespFormer encoder

**Figure 5:**
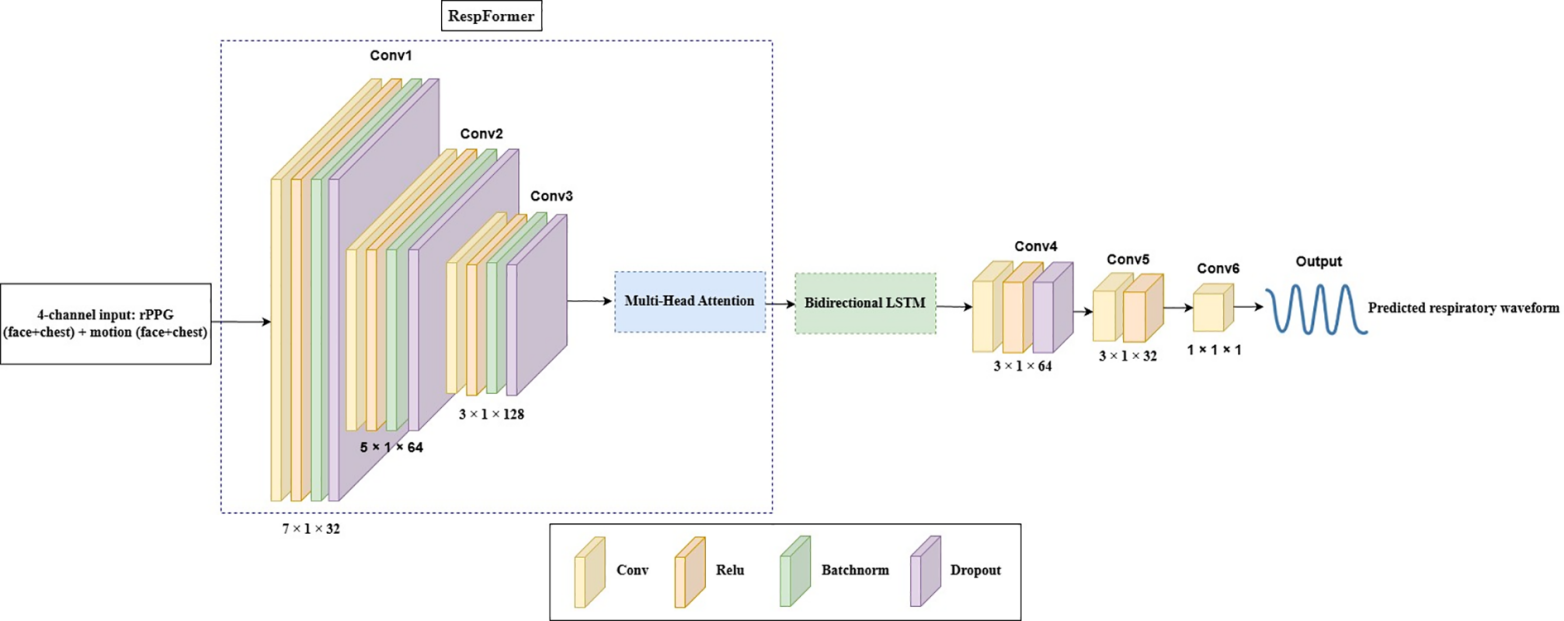
Overall architecture of the DeepRespNet framework. The 4-channel rPPG–motion input is first processed by the RespFormer module to extract multi-scale temporal features. A two-layer BiLSTM then models bidirectional temporal dependencies, and Conv4–Conv6 reconstruct the final respiratory waveform.

The first stage of the framework is the *RespFormer* encoder, which integrates convolutional feature extraction and multi-head self-attention to generate physiologically meaningful representations from the rPPG and motion signals. As illustrated in **Figure 5**, the input sequence is first processed through three one-dimensional convolutional layers (Conv1–Conv3) with progressively decreasing kernel sizes (7, 5, and 3). This hierarchical design enables the network to capture both low-frequency respiratory oscillations and subtle temporal variations. Each convolutional block includes ReLU activation, batch normalization, and dropout to stabilize the training and reduce overfitting. The one-dimensional convolution operation is mathematically expressed in Equation 13:

(13) $$y(t)=\sum_{i=0}^{L-1}w_{i}\cdot x(t-i)+b$$

where *L* denotes the kernel size, *w_i_*represents the learnable weights, and *b* is the bias term.

**Figure 6:**
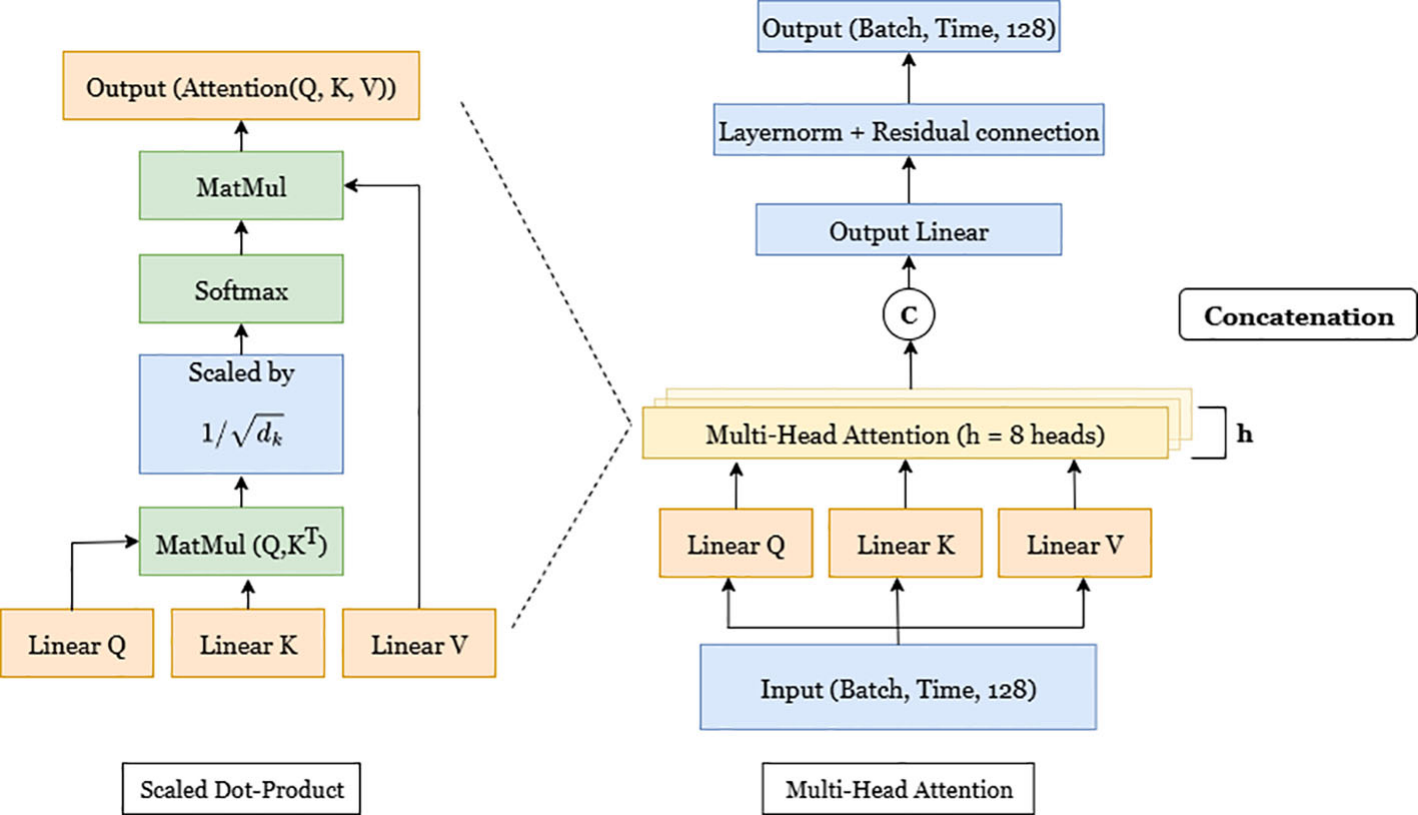
Illustration of the multi-head attention mechanism. The left part shows the scaled dot-product attention, and the right part depicts the multi-head structure with eight parallel heads followed by concatenation and normalization.

The multi-head attention module (**Figure 6**) refines these feature maps by enabling global interactions across the sequence. The attention mechanism (Vaswani et al., 2017) computes pairwise dependencies across the entire sequence, which enables the model to capture long-range temporal relationships. For each attention head, the input is projected into query (*Q*), key (*K*), and value (*V)* representations, and the output is computed using Equation 14 as.

(14) $$\text{Attention}(Q,K,V)=\text{softmax ​}(\frac{QK^{T}}{\sqrt{d_{k}}})V$$

where *d_k_*is the dimension of the key vectors, and the scaling factor $\frac{1}{\sqrt{d_{k}}}$ prevents the softmax function from producing extremely small gradients for large dimensionalities.

Eight attention heads are used to capture multi-scale temporal dependencies, and their outputs are concatenated, linearly transformed, and normalized by a residual connection to stabilize optimization. This mechanism allows the RespFormer to emphasize temporally coherent respiratory cues while suppressing unrelated fluctuations.

#### 2.5.2 BiLSTM module

**Figure 7:**
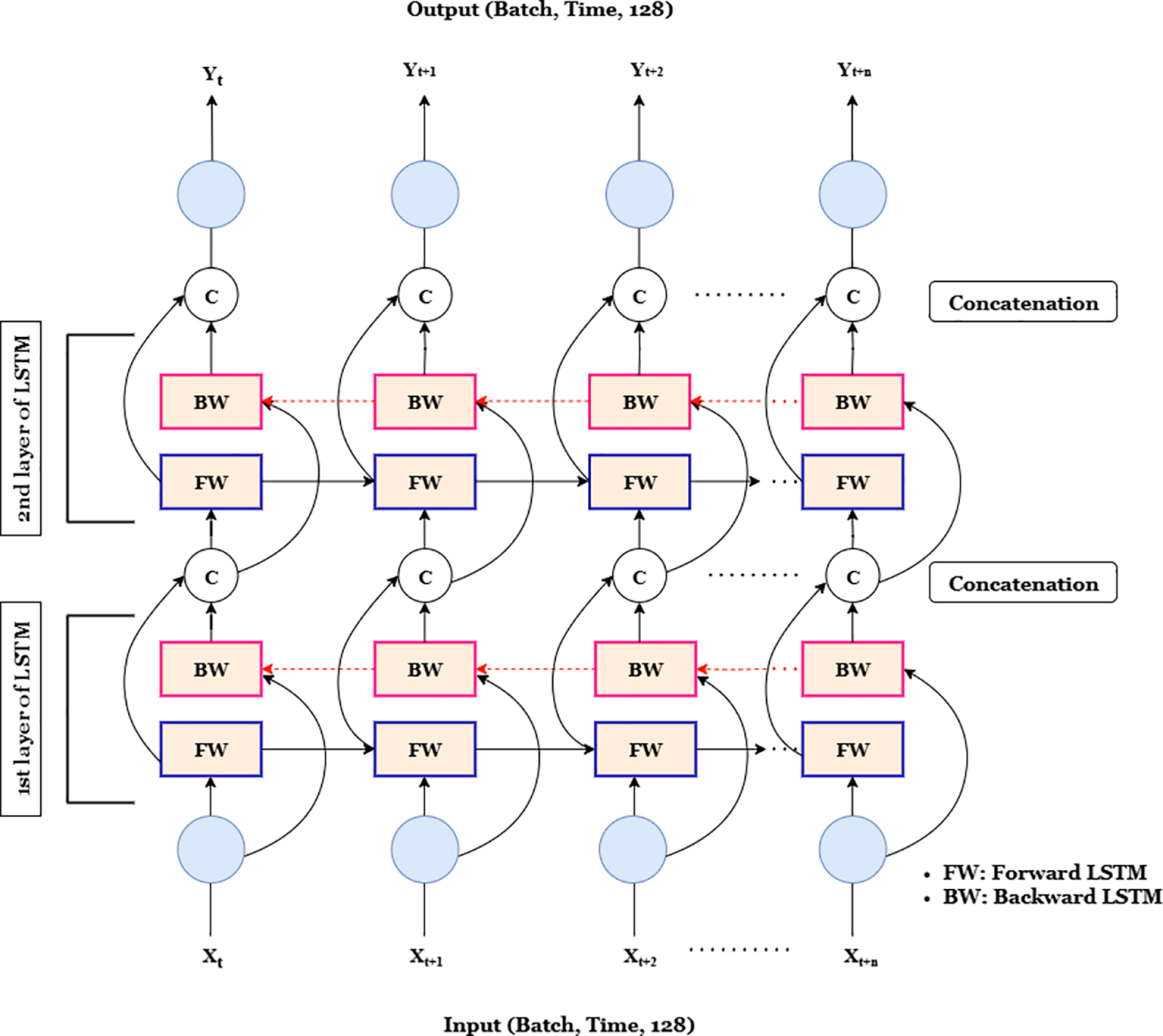
Two-layer BiLSTM architecture showing forward (FW) and backward (BW) flows. Outputs from both directions are concatenated at each timestep to form the final representation.

Following the RespFormer encoder, a two-layer BiLSTM (**Figure 7**) models bidirectional temporal dependencies by leveraging both past and future contexts. Each LSTM unit updates its gates according to Equation 15.

(15) $$\left[f_{t},i_{t},o_{t}]=\sigma (W[x_{t},h_{t-1}]+b\right)$$

The memory cell is then updated using Equation 16.

(16) $$C_{t}=f_{t}⊙C_{t-1}+i_{t}⊙\text{tanh }(W_{c}[x_{t},h_{t-1}]+b_{c})$$

and the hidden state is computed as expressed as in Equation 17.

(17) $$h_{t}=o_{t}⊙\text{tanh }(C_{t})$$

where $f_{t}$, $i_{t}$, and $o_{t}$ denote the forget, input, and output gates, respectively; W and $W_{c}$ represent the learnable weight matrices; b and $b_{c}$ are bias terms.

In the bidirectional configuration, the hidden states from the forward and backward passes are concatenated as shown in Equation 18:

(18) $$h_{t}^{bi}=[h_{t}^{forward};\;h_{t}^{backward}]$$

In the time-unrolled view (**Figure 7**), the input sequence *X_t_,X_t_*_+1_*,…,X_t_*_+_*_n_* represents 128-dimensional feature vectors, forming a tensor of shape (Batch,Time,128). The BiLSTM produces corresponding outputs *Y_t_,Y_t_*_+1_*,…,Y_t_*_+_*_n_* of the same dimension. This bidirectional temporal modeling effectively captures the complete dynamics of the breathing cycle, enhancing temporal coherence and robustness in RR estimation.

**Table 3** summarizes the training configuration and hyperparameters used to optimize the proposed RespFormer–BiLSTM framework. Subject-level *k*-fold cross-validation (*k* = 5) is used, where subjects are split into mutually exclusive folds. In each fold, the model is trained on multiple subjects and tested on unseen subjects, ensuring no subject overlap between each splits. The same evaluation protocol was consistently applied across the public datasets individually.

**Table 3:** Training configuration and hyperparameters of the DeepRespNet system.

Parameter	Value
Evaluation Protocol	Subject-level *k*-fold cross-validation
Number of folds (*k*)	5
Data Split	70% train, 10% validation, 20% test (per fold)
Optimizer	AdamW
Learning Rate (LR)	0.001
Batch Size	32
Epochs	100
Scheduler	Reduce LR on Plateau
Weight Decay	1 × 10^−4^
Dropout	0.2 in attention and BiLSTM layers

The listed hyperparameters were consistently applied in all experiments to maintain uniform training conditions and ensure a fair performance comparison.

### 2.6 Output processing and RR estimation

After the feature extraction and temporal modeling stages illustrated in **Figure 5**, the final stage of the network transforms the learned temporal representations into a continuous respiratory waveform and estimates the RR.

#### 2.6.1 Regression and predicted waveform

The final convolutional layers (Conv4–Conv6 in **Figure 5**) compress the BiLSTM output tensor (Batch,Time,128) into a single-channel temporal waveform through successive one-dimensional convolutions, as represented in Equation 19:

(19) $$Y_{\text{conv}4}\in {\mathbb{R}}^{B\times T\times 64},\;Y_{\text{conv}5}\in {\mathbb{R}}^{B\times T\times 32},\;Y_{\text{conv}6}\in {\mathbb{R}}^{B\times T\times 1}$$

The final convolutional layer produces a one-dimensional temporal output representing the estimated respiratory signal, as expressed in Equation 20:

(20) $$\hat{s}(t)={\text{Conv}}_{1\times 1}(Y_{\text{conv}6})$$

where $\hat{s}(t)$ denotes the predicted respiratory waveform for each input window.

#### 2.6.2 Post-processing of predicted signal

To enhance the signal quality, $\hat{s}(t)$ undergoes detrending and bandpass filtering (0.1–0.6 Hz) to remove baseline drift and isolate respiration-dominant oscillations, as shown in Equation 21:

(21) $${\hat{s}}_{f}(t)={\text{Bandpass}}_{\left[0.1,\;0.6\right]}(\text{Detrend}(\hat{s}(t)))$$

where ${\hat{s}}_{f}(t)$ represents the clean, respiration-dominant signal used for subsequent spectral analysis. This process effectively removes illumination variation, motion artifacts, and slow drift components, ensuring that only respiratory-induced oscillations are preserved.

#### 2.6.3 Spectral analysis and RR estimation

The RR is derived from ${\hat{s}}_{f}(t)$ using a frequency-domain approach based on Welch’s power spectral density (PSD) method (Welch, 1967), as expressed in Equation 22:

(22) $$P(\omega )=\text{Welch}\{{\hat{s}}_{f}(t)\}$$

The Welch’s method was applied to overlapping temporal segments of the respiratory signal rather than the entire sequence. Each segment was processed using a Hamming window to reduce spectral leakage, and adjacent segments were overlapped by 50%. The power spectral density was obtained by averaging the spectra of all segments.

The dominant angular frequency *ω*^∗^ is determined using Equation 23.

(23) $$\omega^{*}=\text{arg }\underset{\omega }{\max}P(\omega )$$

and the RR in BPM is computed using Equation 24.

(24) $$RR=\omega^{*}\cdot \frac{60}{2\pi }$$

Alternatively, RR can be expressed in the discrete frequency domain in terms of the peak frequency component $f_{\text{peak}}$ as shown in Equation 25.

(25) $$RR=f_{\text{peak}}\times 60$$

where $f_{\text{peak}}$ represents the frequency (in Hertz) having the maximum spectral power within the respiratory band.

## 3 Results

### 3.1 Quantitative performance and comparison with existing methods

The performance of the DeepRespNet system was quantitatively evaluated using the mean absolute error (MAE) and root mean square error (RMSE) metrics, which measure the deviation between the estimated and reference RRs. The mathematical formulations of these metrics are given in Equations 26 and 27, respectively:

(26) $$\text{MAE}=\frac{1}{N}\sum_{i=1}^{N}|y_{\text{ref},i}-y_{i}|$$

(27) $$\text{RMSE}=\sqrt{\frac{1}{N}\sum_{i=1}^{N}{\left(y_{\text{ref},i}-y_{i}\right)}^{2}}$$

where $y_{\text{ref},i}$ and $y_{i}$ represent the reference and predicted RRs for the *i*-th sample, respectively, and *N* is the total number of samples considered for the evaluation.

The model performance was evaluated using two public benchmark datasets—UBFC-rPPG (Bobbia et al., 2019) and PURE (Stricker et al., 2014)—and a custom dataset collected for this study. Three evaluation scenarios were analyzed: (1) light skin group, (2) dark skin group, and (3) cross-dataset testing.

An ablation study was also conducted to assess the contribution of each architectural component, including the CNN-only baseline, RespFormer-based architecture, RespFormer with BiLSTM temporal modeling, and the full framework with adaptive preprocessing and multimodal fusion. **Table 4** presents the MAE and RMSE values across all datasets and configurations, demonstrating the progressive improvements achieved through temporal modeling and attention-based fusion.

**Table 4:** Performance comparison and ablation study for the presented method.

Architecture	Dataset	MAE	RMSE
CNN only	UBFC-rPPG	1.21	1.89
	PURE	1.18	1.73
	Custom (Light)	1.11	1.63
	Custom (Dark)	1.37	2.39
RespFormer (CNN + Attention)	UBFC-rPPG	1.07	1.58
	PURE	1.06	1.53
	Custom (Light)	1.03	1.47
	Custom (Dark)	1.25	1.82
RespFormer + BiLSTM	UBFC-rPPG	0.94	1.12
	PURE	0.83	1.06
	Custom (Light)	0.86	1.03
	Custom (Dark)	1.03	1.65
Proposed (Fusion + Adaptive Preprocessing)	UBFC-rPPG	0.62	0.88
	PURE	0.58	0.79
	Custom (Light)	0.45	0.68
	Custom (Dark)	0.80	1.08
	Train on light and test on dark	2.01	3.05
	Train on dark and test on light	1.74	2.76

MAE and RMSE are reported in BPM. Progressive improvements demonstrate the benefit of attention-based encoding, temporal modeling, and adaptive fusion.

To further address the robustness of the proposed framework across varying skin tones, **Table 5** provides a comparison with representative baseline methods, including traditional rPPG approaches (CHROM, POS) and recent deep learning models from the rPPG-toolbox (Liu et al., 2023b). Although these methods are primarily designed for HR estimation, their performance ranges offer a useful reference for evaluating RR estimation capability.

**Table 5:** Comparison of baseline methods for RR estimation.

Method	Dataset	MAE	RMSE
CHROM (de Haan and Jeanne, 2013)	UBFC-rPPG	5.69	6.38
	PURE	5.21	6.22
	Custom (Light)	5.26	6.26
	Custom (Dark)	6.12	6.98
POS (Wang et al., 2016)	UBFC-rPPG	4.91	5.77
	PURE	4.78	5.29
	Custom (Light)	4.90	5.67
	Custom (Dark)	5.12	6.15
DeepPhys (Chen and McDuff, 2018b)	UBFC-rPPG	4.20	4.88
	PURE	3.98	4.26
	Custom (Light)	3.93	4.42
	Custom (Dark)	5.05	6.39
PhysNet (Yu et al., 2019b)	UBFC-rPPG	4.10	4.83
	PURE	3.89	4.36
	Custom (Light)	4.13	5.11
	Custom (Dark)	4.86	6.16
TS-CAN (Liu et al., 2020)	UBFC-rPPG	4.74	5.26
	PURE	4.38	5.06
	Custom (Light)	4.42	5.39
	Custom (Dark)	5.17	6.3
EfficientPhys (Liu et al., 2023a)	UBFC-rPPG	4.0	5.13
	PURE	3.93	4.88
	Custom (Light)	4.13	5.34
	Custom (Dark)	4.98	6.28
DeepRespNet	UBFC-rPPG	0.62	0.88
	PURE	0.58	0.79
	Custom (Light)	0.45	0.68
	Custom (Dark)	0.80	1.08

Baseline methods are adopted from prior studies originally designed for HR estimation. These methods here provide a comparative evaluation for RR estimation on both public datasets and the proposed custom dataset.

In addition, to provide a comparative analysis with the existing deep learning-based RR-based models, **Table 6** summarizes overall performance of these methods with the proposed architecture. While these studies use different datasets and evaluation settings, they provide an insight when compared with the proposed framework that achieves better accuracy with stable performance across varying skin tones.

**Table 6:** Comparison of deep learning-based noncontact RR estimation methods.

Reference methodology	Dataset	MAE (BPM)	
(Ghezzi et al., 2024)	Clifford neural layers for geometric modeling	SCAMPS, COHFACE, BP4D+	0.83
(Suriani et al., 2022)	2D spatiotemporal CNN	Custom	1.53
(Saryanto and Andriyani, 2024)	CNN with magnified components video	Custom	1.70
(Ren et al., 2022)	Multitask convolutional attention network	COHFACE	2.05
(Akamatsu et al., 2023)	Self-supervised learning with data augmentation	UBFC-rPPG, Custom	0.95
Proposed	DeepRespNet	Custom	0.45– 0.80

All reported values represent MAE in BPM. It should be noted that dataset characteristics, acquisition conditions, and evaluation protocols vary between studies; therefore, this comparison serves as an indicative benchmark rather than a direct equivalence test. However, the proposed method shows consistent accuracy across the tested skin tone groups.

### 3.2 Experimental analysis

**Figure 8:**
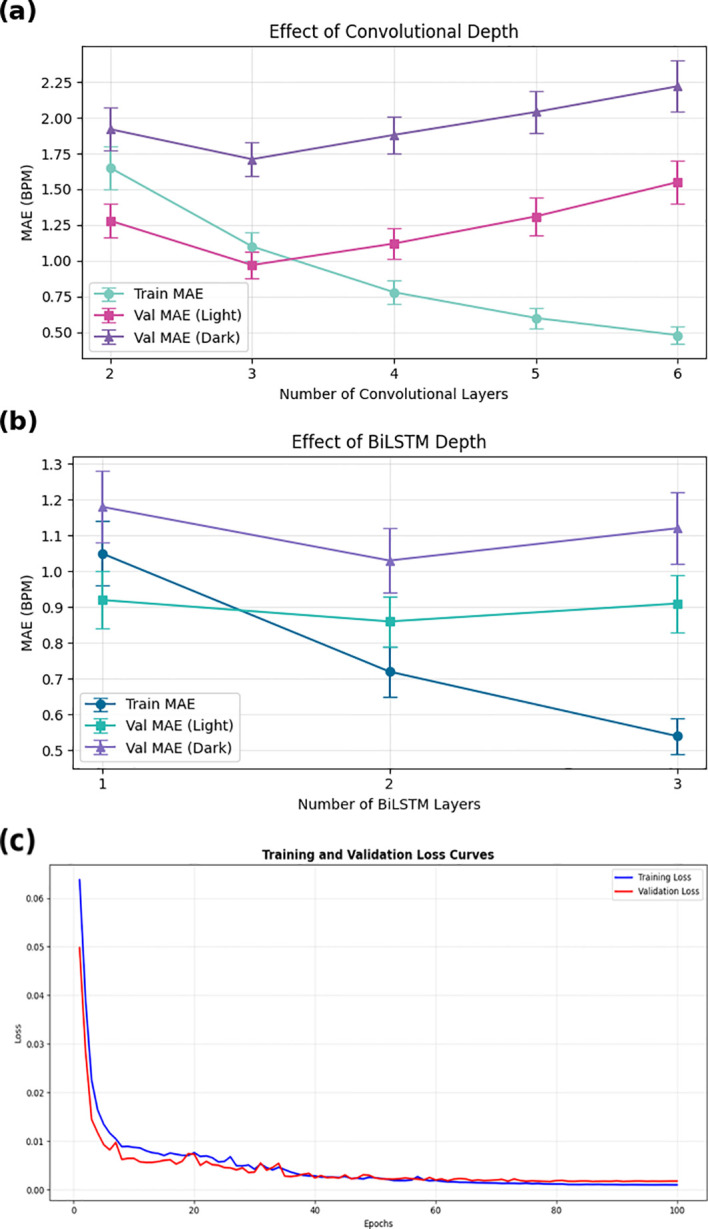
Network depth analysis and training stability validation. **(a)** CNN performance peaks at three layers before overfitting increases, showing optimal validation MAE for both light and dark skin groups. **(b)** BiLSTM performance is optimal at two layers, with deeper configurations reducing generalization. **(c)** Training and validation loss convergence illustrating stable optimization and strong generalization, with convergence achieved after approximately 40 epochs.

In addition to the ablation experiments, further analyses were conducted to justify the selected network depth and to examine the robustness of the proposed framework under challenging respiratory conditions. As illustrated in **Figure 8a**, increasing the number of convolutional layers monotonically reduces the training error; however, the validation MAE exhibits a clear U-shaped pattern. Performance improves when increasing the depth from two to three layers, whereas deeper configurations (four to six layers) result in increased validation error despite lower training loss, indicating overfitting. Therefore, a three-layer convolutional encoder was selected for the RespFormer. A similar trend is observed for the BiLSTM depth (**Figure 8b**). Transitioning from one to two layers consistently decreases the validation MAE whereas adding a third layer yields higher error due to over smoothing and reduced generalization across both skin tone groups. These observations validate the architectural choices used in the final RespFormer–BiLSTM framework.

The proposed network’s training stability was validated by analyzing loss convergence behavior for both training and validation phases, as illustrated in **Figure 8c**. The loss decreases rapidly during the early epochs and stabilizes after approximately 40 epochs, indicating effective learning and minimal overfitting. This convergence pattern confirms stable learning dynamics and reliable generalization to unseen data across different evaluation scenarios.

**Figure 9:**
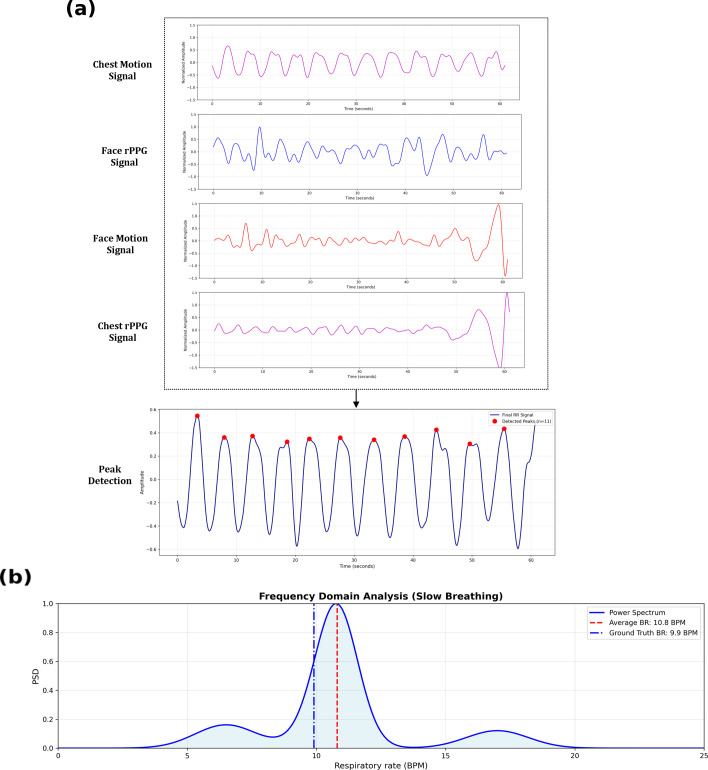
Time-frequency analysis of slow breathing patterns. **(a)** Time-domain visualization showing four input modalities (face and chest rPPG and motion) fused into a composite respiratory waveform with accurate peak detection despite slow, deep breathing. **(b)** Frequency-domain representation via Welch’s PSD confirming precise identification of the dominant respiratory frequency.

A subset of participants in the dataset exhibited slow, deep breathing patterns, and thus this subset was used in an evaluation of the model under low-frequency respiratory conditions. As illustrated in **Figure 9a**, the four input modalities—face rPPG, chest rPPG, face motion, and chest motion—exhibited subtle oscillations that are challenging to interpret individually. After fusion and temporal filtering, the composite reconstructed respiratory waveform demonstrated a clearly periodic structure, and the proposed framework accurately identified each respiratory peak despite the reduced breathing rate. The corresponding frequency-domain representation in **Figure 9b**, obtained using Welch’s power spectral density analysis, confirms precise localization of the dominant respiratory frequency and yields an average MAE of 0.9 BPM.

Although the number of slow-breathing samples was relatively limited, this experiment highlighted the model’s capacity to generalize across different RRs. Expanding the dataset with additional slow-breathing recordings could further enhance the network’s robustness and improve low-frequency signal discrimination in future studies.

**Figure 10:**
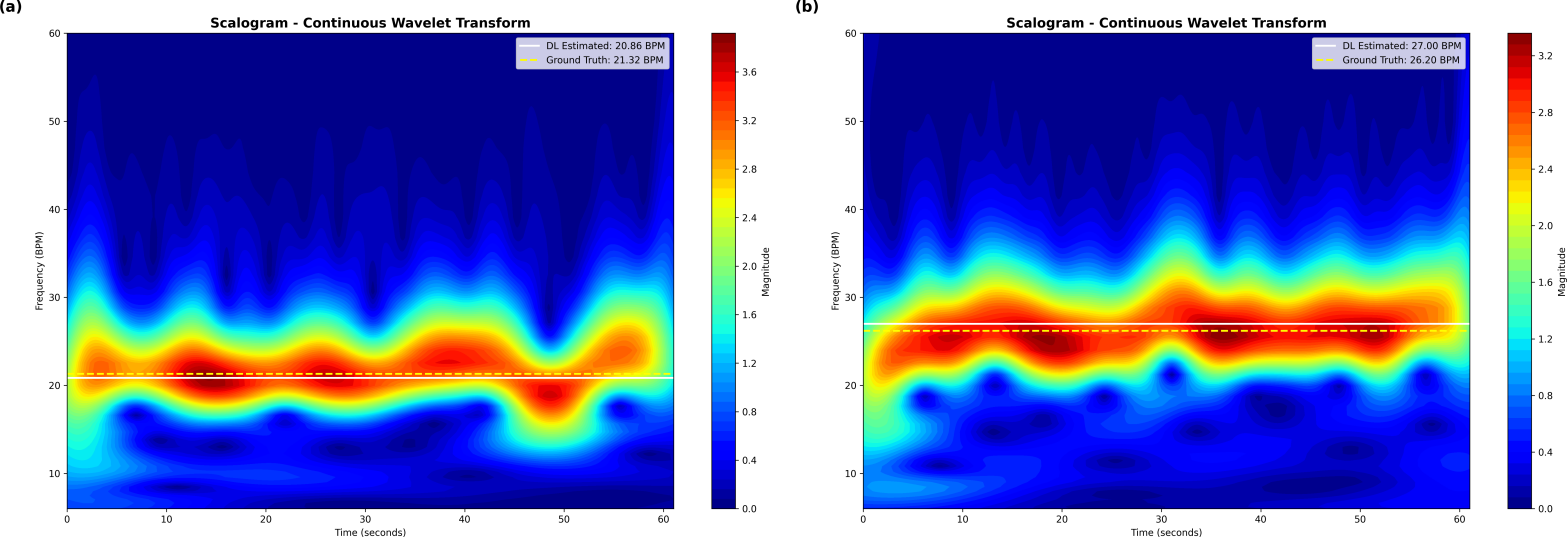
Scalogram representations of respiration for **(a)** Light-skinned subjects and **(b)** Dark-skinned subjects obtained using CWT.

To further examine the spectral characteristics of the reconstructed respiratory signals, continuous wavelet transform (CWT) analysis was performed. As illustrated in **Figure 10**, the scalograms highlight the model’s ability to preserve dominant respiratory oscillations across both light- and dark-skinned subjects. The presence of stable high-energy ridges aligned with the ground-truth respiratory frequencies indicates that the learned representations are both temporally coherent and spectrally consistent. The similarity of scalogram patterns across skin tones further suggests that the fusion of rPPG- and motion-derived cues helps maintain stable respiratory representations under varying optical conditions.

**Figure 11:**
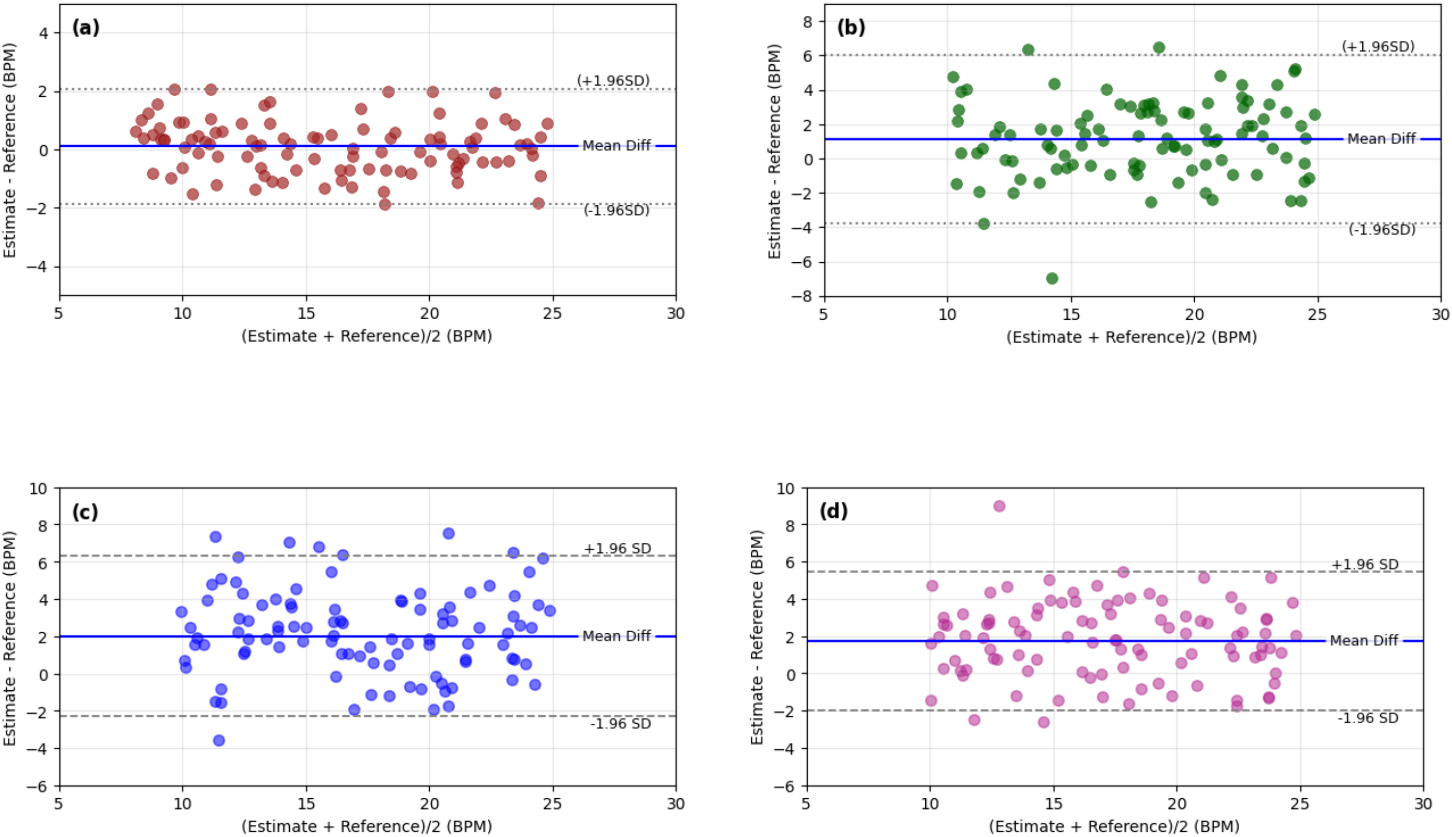
Bland–Altman agreement analysis for within-dataset and cross-dataset evaluation. **(a)** Lightskinned subjects. **(b)** Dark-skinned subjects. Both show tight clustering within 95% limits. **(c)** Train on light and test on dark shows broader dispersion. **(d)** Train on dark and test on light demonstrates tighter agreement, confirming improved generalization from training on challenging conditions.

Agreement between the estimated and reference respiratory rates was further evaluated using Bland–Altman analysis, as illustrated in **Figures 11a** and **b**. For the light-skin group, the mean difference between the estimated and reference measurements is close 0 BPM, with limits of agreement spanning −2 to +2 BPM, indicating minimal systematic bias. For the dark-skin group, the mean difference is approximately +1.5 BPM, with limits of agreement ranging from −4 to +6 BPM. These results suggest that the proposed framework does not consistently overestimate or underestimate RR across the evaluated samples, though a slight positive bias is present for darker skin tones.

The observed limits of agreement are consistent with the level of agreement typically reported in camera-based respiratory monitoring studies (Nishidate et al., 2022). The narrower dispersion for the light-skin group can be attributed to stronger rPPG signal quality, while the wider spread for the dark-skin group reflects the reduced optical signal amplitude typically associated with lower reflectance in darker skin tones (Bondarenko et al., 2025). Notably, the asymmetric limits in the dark-skin group (−4 to +6 BPM) indicate a mild tendency toward positive estimation error, consistent with the increased signal uncertainty in this condition.

Notably, the estimation error does not exhibit a systematic dependence on the RR magnitude along the x-axis in either group, confirming the absence of proportional bias. The error distribution remains uniform across the evaluated respiratory range, indicating that the proposed framework maintains consistent estimation performance for both lower and higher breathing frequencies.

Cross-dataset evaluation results are presented in **Figures 11c, d**. When the model is trained on light-skinned subjects and evaluated on dark-skinned subjects, the Bland–Altman plots show a broader spread around the mean difference, reflecting increased variability associated with weaker rPPG signal strength. Conversely, training on dark-skinned subjects and testing on light-skinned subjects results in tighter clustering and smaller deviations. This behavior suggests that training under more challenging signal conditions can improve the robustness and generalization capability of the model when applied to more favorable signal environments.

**Figure 12:**
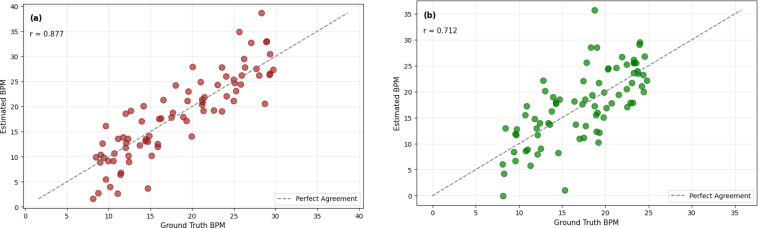
Scatter plots showing the correlation between estimated and reference respiratory rates for **(a)** Light-skinned subjects and **(b)** Dark-skinned subjects.

The scatter plots in **Figure 12** provide a complementary visualization of the relationship between the estimated and reference RRs. The near-linear trends observed across both skin-tone groups indicate that the proposed model captures the underlying respiration dynamics with consistent predictive behavior across the evaluated samples.

These analyses collectively demonstrate that the proposed framework achieves a balanced tradeoff between model complexity, robustness to low-frequency respiration, and stable optimization behavior, thereby supporting its suitability for practical non-contact respiratory monitoring under diverse physiological conditions.

## 4 Discussion

This study demonstrates that camera-based RR estimation can achieve clinically relevant accuracy across diverse populations using the proposed DeepRespNet framework. The experimental results indicate consistent RR estimation across varying skin tones and dynamic conditions. The key contribution of the proposed framework is the integration of multiple respiration-related cues through the RespFormer–BiLSTM architecture. By combining rPPG-derived physiological signals with motion-based features capturing subtle head and chest displacements, the proposed model leverages complementary sources of respiratory information. This multimodal fusion enables the model to maintain stable pulse related signal representations even when one modality is partially degraded due to illumination variations, skin tone differences, or subject motion. Thereby, it improves the robustness of the proposed approach when compared to conventional single-modality camera-based respiration monitoring systems.

Despite the promising results observed across multiple datasets and evaluation settings, several limitations of the present study should be acknowledged. First, although the proposed framework demonstrates robustness across different skin tones, its performance degrades under cross-dataset scenarios, particularly when models trained on higher-quality signals are evaluated on lower-SNR conditions. This indicates residual sensitivity to domain shifts arising from variations in illumination, camera characteristics, and subject motion. Second, the evaluation was primarily conducted under controlled or static environments; abrupt body movements and severe illumination changes were not extensively represented in the dataset. Additionally, the number of slow and irregular breathing samples was relatively limited, which may constrain the model’s generalization to extreme respiratory patterns.

Future work will focus on improving robustness under more challenging real-world conditions. Incorporating explicit motion compensation strategies and domain-adaptive learning techniques may help reduce cross-dataset performance degradation. The integration of additional sensing modalities, such as infrared or thermal imaging, could further enhance performance under low-light or motion-intensive scenarios. Moreover, expanding the dataset to include a wider range of breathing patterns, environmental conditions, and subject demographics will be essential for improving generalization and clinical reliability. In future studies, real-time respiratory data will be collected from hospital environments to further validate the proposed framework and construct a more diverse dataset encompassing various patient conditions and clinical scenarios. Such data will also enable the investigation of respiration-related abnormalities and support the development of extended models for early detection of respiratory disorders in conditions such as sleep disorders, postoperative recovery, and chronic pulmonary disease. Finally, real-time implementation and large-scale validation in telemedicine or home-monitoring settings will be explored to assess the practical applicability of the proposed framework for continuous, contactless respiratory monitoring.

## Data Availability

The raw data supporting the conclusions of this article will be made available upon request to the corresponding author.
